# Hyaluronic acid production by *Streptococcus zooepidemicus *in marine by-products media from mussel processing wastewaters and tuna peptone viscera

**DOI:** 10.1186/1475-2859-9-46

**Published:** 2010-06-14

**Authors:** José A Vázquez, María I Montemayor, Javier Fraguas, Miguel A Murado

**Affiliations:** 1 Grupo de Reciclado y Valorización de Materiales Residuales (REVAL) Instituto de Investigacións Mariñas (CSIC)., r/Eduardo Cabello, 6. Vigo-36208. Galicia - Spain; 2 Dilsea S.L., Porto Pesqueiro de Vigo, dársena 3., Vigo-36202. Galicia - Spain

## Abstract

**Background:**

Hyaluronic acid is one of the biopolymers most commonly used by the pharmaceutical industry. Thus, there is an increasing number of recent works that deal with the production of microbial hyaluronic acid. Different properties and characteristics of the fermentation process have been extensively optimised; however, new carbon and protein sources obtained from by-products or cheap substrates have not yet been studied.

**Results:**

Mussel processing wastewater (MPW) was used as a sugar source and tuna peptone (TP) from viscera residue as a protein substrate for the production of hyaluronic acid (HA), biomass and lactic acid (LA) by *Streptococcus zooepidemicus *in batch fermentation. Commercial medium formulated with glucose and tryptone was used as the control. The parametric estimations obtained from logistic equations and maintenance energy model utilized for modelling experimental data were compared in commercial and low-cost media. Complete residual media achieved high production (3.67, 2.46 and 30.83 g l^-1 ^of biomass, HA and LA respectively) and a high molecular weight of HA (approximately 2500 kDa). A simple economic analysis highlighted the potential viability of this marine media for reducing the production costs by more than 50%.

**Conclusions:**

The experimental data and mathematical descriptions reported in this article demonstrate the potential of media formulated with MPW and TP to be used as substrates for HA production by *S. zooepidemicus*. Furthermore, the proposed equations accurately simulated the experimental profiles and generated a set of interesting parameters that can be used to compare the different bacterial cultures. To the best of our knowledge, this is the first work in which a culture media formed by marine by-products has been successfully used for microbial HA production.

## Background

Hyaluronic acid (HA) is a linear and high molecular mass polymer formed by repeating disaccharide units of N-acetyl-D-glucosamine and D-glucuronic linked by β(1-3) and β(1-4) glycosidic bonds. Because its physicochemical and biological properties, such as lubricity, viscoelasticity, water holding capacity and biocompatibility, HA has numerous and increasing applications in food, cosmetic and clinical areas such as plastic surgery, treatment of arthritis, major burns and intra-ocular surgery [1,2]. This glycosaminoglycan has traditionally been extracted from animal tissues such as synovial fluid, rooster combs, cartilage, vitreous humour and umbilical cords [3,4]; however, fermentative HA production by *Streptococcus *generates yields with higher concentrations of HA at lower costs and with more efficient downstream processes [5-7]. Among the strains of this bacteria, *S. zooepidemicus *is one of the most commonly used [6,7]. The strains of this bacteria are facultative anaerobes, but they are also aerotolerant, catalase-negative and have fastidious nutrient requirements with respect to organic nitrogen [5,8].

Although several strategies have been reported for increasing microbial HA, including pH-gradient stress [8], continuous culture [9], lysozyme or hyaluronidase addition [10,11], agitation and aeration conditions [12-14], medium optimisation [15], the type of bioreactor [16], effect of aminoacids and mineral salts [17,18] and fed-batch operation [19], there is almost no studies of new sources of sugars and proteins from organic waste materials in order to reduce both production costs and pollution problems. More than 80% of these costs are due to these nutrients (sugars and proteins) and commercial formulations are not an economical resource for industrial production of HA.

Peptones obtained from fish viscera residues have been found to be an excellent substrate for different microbial processes [20-25]. Recently, we studied the appropriateness of two marine peptones for the production of lactic and hylauronic acids [26]. Furthermore, mussel processing wastewaters (MPW), a residual material rich in glycogen obtained from canning companies, has been used in several bioproductions, including the production of gibberellins [27], amylase [28], bacteriocins [29-31], glucose oxidase [32] and citric acid [33]. From an environmental point of view, both residues generate serious pollution problems on Galician coasts (NW, Spain) as they are produced in large volumes and have a high organic load, which makes their depuration extremely difficult. The European Union guidelines about this problem are based on the development and implementation of an efficient and integral waste management and valorisation processing in order to obtain zero-wastes, zero-discharges and zero-pollution.

The main aim of this work was to investigate the fermentative capacity of culture media formulated with MPW and marine peptones, obtained from two different, highly polluting marine by-products, in order to replace the expensive commercial sources of carbohydrates and proteins usually used in hyaluronic acid production by *S. zooepidemicus*. The kinetic parameters obtained from a modified logistic equation were successfully used to compare accurately the corresponding metabolite productions.

## Results

To study the appropriateness of marine by-product substrates for the production of HA by *S. zooepidemicus*, glucose and commercial peptone in the control medium were replaced by MPW and peptone from tuna viscera by-products respectively. All carbohydrate sources were employed at a concentration of 50 g l^-1^. Cultures were grown in the nutritive formulations described in Table 1.

**Table 1:** Composition of culture media for *Streptococcus zooepidemicus *(g l^-1^).

Compounds	A	B	C	D
Glucose	50.0	50.0	-	-
Glycogen (from MPW)	-	-	50.0	50.0
Yeast extract	5.0	5.0	5.0	5.0
Tryptone	15.0	-	15.0	-
KH_2_PO_4_	2.0	2.0	2.0	2.0
K_2_HPO_4_	2.0	2.0	2.0	2.0
MgSO_4_.7H_2_O	0.5	0.5	0.5	0.5
(NH4)_2_SO_4_	0.5	0.5	0.5	0.5
Polystyrene (Mw = 990 kDa)	0.015	0.015	0.015	0.015
Tuna-peptone protein (Lowry)	-	8.0	-	8.0

A: Complex medium (used as control).

B: Medium formulated with glucose as carbohydrate-substrate and protein from tuna viscera wastes.

C: Formulation containing commercial tryptone and mussel processing wastewaters (MPW).

D: Medium with glycogen from MPW and peptone from viscera by-products of tuna.

**Figure 1:**
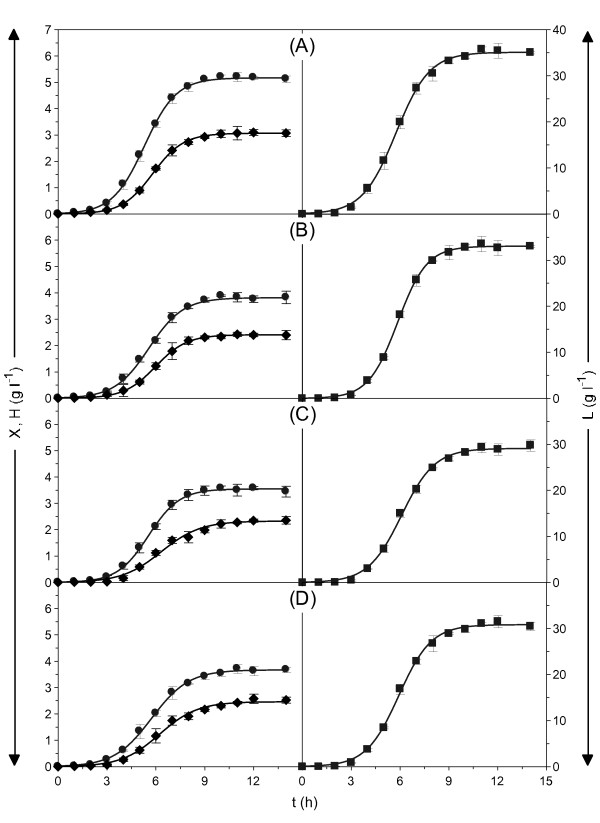
**Metabolic productions from *Streptococcus zooepidemicus *batch-cultures in the media specified in Table 1**. A: complex medium with all commercial chemicals, B: complex medium replacing tryptone by tuna peptone from viscera residues, C: complex medium replacing glucose by MPW, D: medium formulated with tuna peptone and MPW. Solid lines represent the fitting functions corresponding to the experimental data (points) according to the equations (1-4). X: biomass (black circle); H: hyaluronic acid (black diamond); L: lactic acid (black square). The error bars showed in the plots are the confidence intervals of independent experiments (α = 0.05, n = 2).

**Figure 2:**
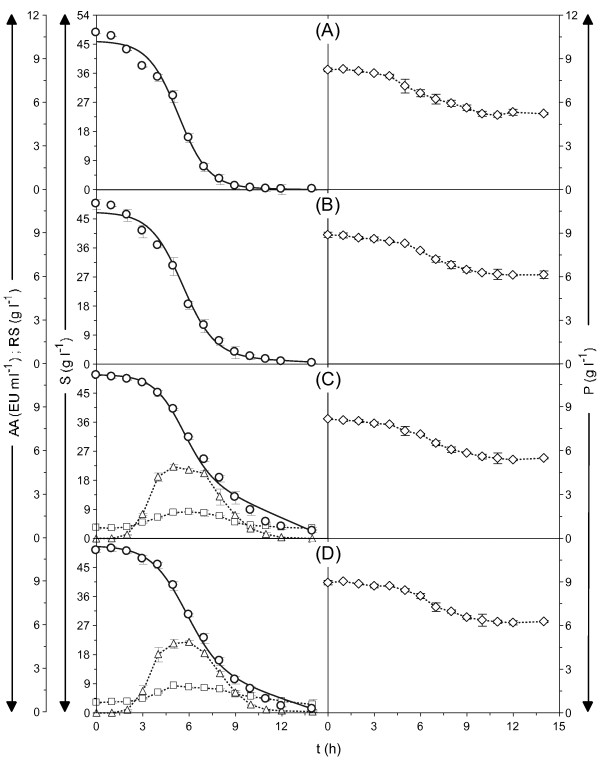
**Substrate consumptions from *Streptococcus zooepidemicus *batch-cultures in the media specified in Table 1**. S: carbohydrate substrate (white circle); AA: total amylolytic activity (white triangle); RS: reducing sugars (white square); P: proteins (white diamond). The rest of notations are similar to the previously described in Figure 1.

Figures 1 and 2 show *S. zooepidemicus *growth overtime, the metabolite productions (LA and HA) and the concomitant substrate consumptions (protein and sugars) during fermentation in the four media. Moreover, in the culture broths formulated with MPW as the carbon source, the total amylolytic activity and reducing sugar concentration were also measured. Sigmoid profiles for production and uptake were obtained for all cases. The numerical values of the kinetic parameters calculated by fitting the experimental data to the mathematical equations described previously, as well as their corresponding statistical analysis, are summarised in Table 2. According to these results, medium A had the highest maximum biomass production (*X*_*m *_= 5.17 ± 0.06 g l^-1 ^h^-1^) maximum growth rate (*v*_*x *_= 1.32 ± 0.08 g l^-1^) and shortest lag phase (*λ*_*x *_= 3.33 ± 0.14 h). In the rest of the media, 25% less biomass was produced than in the control, but there were no significant differences between media B, C or D.

**Table 2:** Parametric estimations obtained from fitted experimental data to the equations (1-6).

VARIABLES	MEDIUM A	MEDIUM B	MEDIUM C	MEDIUM D
BIOMASS (*X*)	values ± CI	values ± CI	values ± CI	values ± CI

*X*_*m *_(g l^-1^)	5.17 ± 0.06	3.81 ± 0.08	3.55 ± 0.05	3.67 ± 0.06
*v*_*x*_(g l^-1 ^h^-1^)	1.32 ± 0.08	0.91 ± 0.09	0.93 ± 0.06	0.81 ± 0.06
*λ*_*x *_(h)	3.33 ± 0.14	3.48 ± 0.23	3.65 ± 0.14	3.46 ± 0.18
*p*-value	<0.001	<0.001	<0.001	<0.001
r (obs-pred)	0.999	0.999	0.999	0.999

HYALURONIC ACID (*H*)	values ± CI	values ± CI	values ± CI	values ± CI

*H*_*m *_(g l^-1^)	3.07 ± 0.03	2.41 ± 0.02	2.33 ± 0.10	2.46 ± 0.09
*v*_*h *_(g l^-1 ^h^-1^)	0.82 ± 0.04	0.63 ± 0.03	0.49 ± 0.09	0.53 ± 0.08
*λ*_*h *_(h)	3.93 ± 0.10	4.07 ± 0.10	3.93 ± 0.48	3.90 ± 0.38
*p*-value	<0.001	<0.001	<0.001	<0.001
r (obs-pred)	0.999	0.999	0.997	0.998

LACTIC ACID (*L*)	values ± CI	values ± CI	values ± CI	values ± CI

*L*_*m *_(g l^-1^)	35.12 ± 0.56	33.04 ± 0.32	29.11 ± 0.55	30.83 ± 0.54
*v*_*l *_(g l^-1 ^h^-1^)	8.53 ± 0.67	9.24 ± 0.49	7.12 ± 0.65	7.73 ± 0.68
*λ*_*l *_(h)	3.69 ± 0.18	4.07 ± 0.11	4.04 ± 0.21	3.93 ± 0.20
*p*-value	<0.001	<0.001	<0.001	<0.001
r (obs-pred)	0.999	0.999	0.999	0.998

SUBSTRATE (*S*)	values ± CI	values ± CI	values ± CI	values ± CI

*S*_0 _(g l^-1^)	45.81 ± 1.78	46.89 ± 1.74	50.63 ± 2.06	51.47 ± 1.60
*Y*_*x*/*s *_(g g^-1^)	0.11 ± 0.01	0.09 ± 0.01	0.11 ± 0.02	0.10 ± 0.01
*m*_*e *_(g g^-1 ^h^-1^)	0.01 (NS)	0.06 (NS)	0.58 ± 0.16	0.43 ± 0.13
*p*-value	<0.001	<0.001	<0.001	<0.001
r (obs-pred)	0.997	0.997	0.995	0.997

YIELDS (*Y*)	values ± CI	values ± CI	values ± CI	values ± CI

*Y*_*h*/*x *_(g g^-1^)	0.58 ± 0.02	0.62 ± 0.02	0.61 ± 0.02	0.65 ± 0.02
*p*-value	<0.001	<0.001	<0.001	<0.001
r (obs-pred)	0.995	0.997	0.988	0.995
*Y*_*l*/*x *_(g g^-1^)	6.61 ± 0.22	8.63 ± 0.24	7.90 ± 0.22	8.43 ± 0.24
*p*-value	<0.001	<0.001	<0.001	<0.001
r (obs-pred)	0.996	0.998	0.995	0.999

CI: confidence intervals (α = 0.05).

*p*-value from F-Fisher test (α = 0.05).

r = correlation coefficient between observed and predicted data.

NS: not significant

Similar results were obtained for HA and LA production. Broth A was the best option for both chemicals in terms of the *H_m_*, *L_m_*, *v_h _*and *v_l _*values. However, the residual media were excellent alternatives for producing these compounds, and more than 30 and 2.4 g l^-1 ^of LA and HA respectively were obtained in medium D.  The yields (*Y_x/s_*, *Y_h/x _*and *Y_l/x_*) in media prepared with by-products were the same or superior to those obtained in the commercial medium. The cultures carried out using glycogen from MPW as the carbohydrate source showed that *S. zooepidemicus *is capable of producing extracellular amylase and intake the glucose that this enzyme liberates in order to be metabolised (Figure 2C and 2D).

From a statistical viewpoint, all fittings were highly satisfactory (Table 2) and experimental data were adequately described by the equations proposed (Figures 1 and 2). The mathematical expressions were consistent (Fisher's F-test) and the parametric estimations were significant (Student's t-test). Furthermore, all the values predicted in the non-linear adjustments produced high coefficients of linear determination with the observed values (r ≥ 0.995).

**Figure 3:**
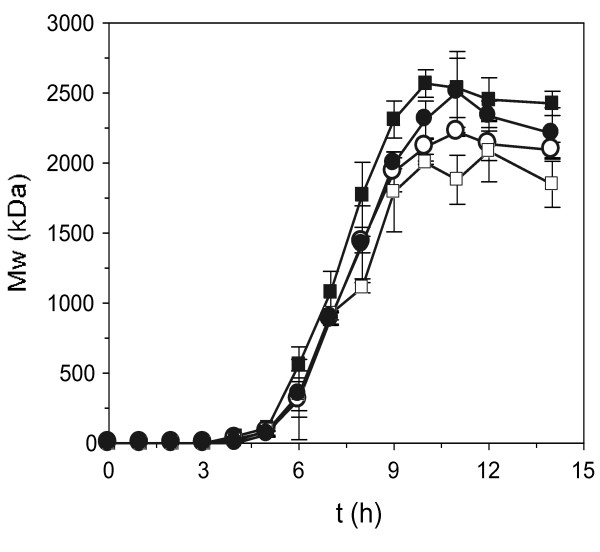
**Molecular weight (*M*_*w*_) profiles of hyaluronic acid obtained in the cultures from Figure 1**. Medium A (white circle), medium B (black square), medium C (white square), medium D (black circle). The error bars showed in the plots are the confidence intervals of independent experiments (α = 0.05, n = 2).

Figure 3 depicts the kinetic profiles of the molecular weight of HA. In all media tested, more than 2000 kDa of polymer were obtained after approximately 9 h. As it can be observed, sigmoid trends with a small drop at 12 h were detected. This fall at the end of the time course can be easily justified by the hyaluronidase action that catalyses the hydrolysis of glycoside bonds in HA, which occurs in the asymptotic phase when biomass production is finished. The largest HA molecules were produced in media B and D, and the smallest glycosaminoglycan molecules were produced in medium A.

**Figure 4:**
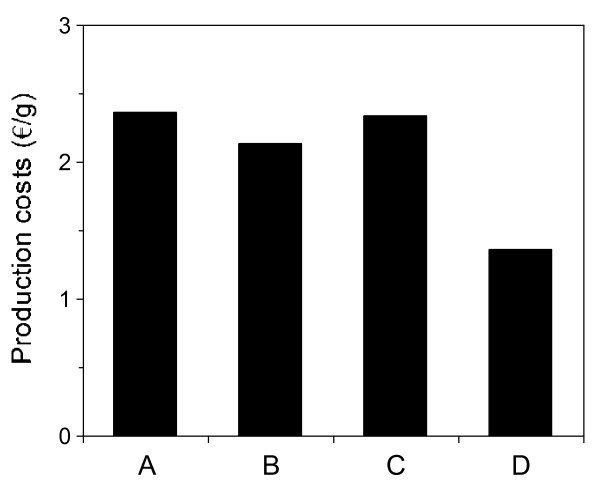
**Cost of hyaluronic acid production in the media studied**. Results are given as €-costs per g of maximum hyaluronic acid produced.

Figure 4 represents the comparative cost of HA production in the different media evaluated. This calculation was carried out using the commercial prices (in €) of the nutrients specified previously (Table 1) and the values of *H_m _*(calculated as the maximum total mass obtained in each bioreactor) summarised in Table 2. It is clear that all media formulated with marine residues achieved lower costs. Furthermore, it is especially remarkable that medium D, composed with MPW as the carbon source and TP as the organic nitrogen source, reduced the price of producing HA by more than 50%.

## Discussion

In recent years there has been increasing interest in microbial HA production by *Streptococcus *and genetically modified bacteria [13,34]. The strategies and methods for improving HA fermentation have been focused on optimising culture conditions and the associated metabolic fluxes [18,35]. However, the possibility of using residual sources of carbon and organic nitrogen from food by-products in order to reduce the price of HA production is still unexplored. In a previous work, we confirmed that marine peptones from shark and ray viscera residues could be used to obtain high molecular weight HA using fed-batch fermentations [26]. It is well known that *Streptococcus *sp. has fastidious nutrient and complex organic nitrogen requirements [36]. The results of *H_m _*with tuna peptone evaluated in the present manuscript showed that tuna peptone has similar nutritive properties (2.46 g l^-1^) to those supplied by commercial tryptone. In addition, the HA production, kinetic profiles and the *M_w _*characteristics of HA were similar to those reported for batch cultures and synthetic media by Armstrong and Johns [37] and Don et al. [38].

Zhang et al. [15] are the only authors who have studied the potential of *S. zooepidemicus *growth and HA production on a polysaccharide substrate. These authors reported excellent yields of HA produced by a *S. zooepidemicus *mutant obtained by successive generation using soluble starch as the carbon source. To our knowledge, the present work is the first article that focuses on HA production with a glycogen substrate from marine wastewater materials (MPW). This liquid residue is generated in great quantities in canning factories and has a negative environmental impact on the Galician Rías (NW, Spain) marine ecosystem, which is of great ecological wealth and very sensitive to contamination. The results obtained for the total amylolytic activity and the corresponding reducing sugars that are liberated confirmed that *S. zooepidemicus *produces extracellular amylase. The maximum activity was achieved at approximately 6 h (Figure 2C and 2D).

In relation to modelling, several mathematical equations have recently been developed for fitting *S. zooepidemicus *growth, HA production and substrate consumption [19,38,39]. The different proposals include two-compartment models [40], neural networks [41], empirical equations from response surfaces [15] and unstructured mathematical models [19,38]. In most cases, logistic and Monod equations - with or without inhibitory terms - were chosen to describe biomass, and growth-associated product formation was used to simulate HA kinetics. Our approach was based on reparameterised logistic models (for growth, HA and LA productions) that consistently describe the experimental profiles and provide a set of significant parameters (maximum productions, lag phases and maximum production rates) for comparing the fermentation media tested. For sugar consumption, a maintenance energy equation was used to fit numerical data and to calculate the production yields. Figures 1 and 2 and Table 2 show the accuracy and statistical robustness of this proposal.

## Conclusions

The most important economic factor in the production of hyaluronic acid is the cost of the complex media. In this sense, the present work demonstrates the excellent viability of a medium formulated with mussel processing wastewaters and peptones obtained from tuna viscera by-products for HA produced by *S. zooepidemicus*. In this medium, productions of 3.67, 2.46 and 30.83 g l^-1 ^of biomass, HA and LA respectively were achieved with a high molecular weight of HA (approximately 2500 kDa). In addition, the manufacturing costs were reduced by more than 50%. Furthermore, all the equations defined not only fit the experimental profiles well but can also be used, as in the present assessment, for comparative purposes in order to optimise the culture medium and experimental conditions for HA microbial production.

## Methods

### Microorganism and fermentation broths

The hyaluronic acid-producing strain used was *Streptococcus equi *subsp. *zooepidemicus *ATCC 35246. Stock cultures were stored at -80°C in complex medium (defined in Table 1) with 25% glycerol. All inocula were prepared following the methodology reported by Vázquez et al. [26].

MPW, produced as a by-product of the mussel-cooking process, was firstly concentrated by means of ultrafiltration membranes with cut-off at 100 kDa until approximately 60 g l^-1 ^of the total sugar concentration (glycogen), according to the methods previously described in detail [42,43]. The initial composition of MPW was: 60.54 g l^-1 ^of total sugars, 1.54 g l^-1 ^of Lowry protein and 0.40 g l^-1 ^total nitrogen.

Solutions of marine peptones from yellowfin tuna viscera (*Thunnus albacares*) were prepared following the operations specified in [44]. The initial composition of tuna peptone (TP) was: 48.32 g l^-1 ^of Lowry protein, 3.11 g l^-1 ^of total sugars and 11.08 g l^-1 ^total nitrogen.

Table 1 shows the composition of the culture media. Yeast extract and tryptone were provided by Cultimed (Panreac Química, Spain) and polystyrene and glucose by Sigma (St. Louis, MO, USA). The protein concentration in the residual media was established by replacing the Lowry protein level in the tryptone (15 g l^-1^) used for the commercial media. MPW was diluted with TP solution and distilled water until approximately 50 g l^-1 ^total sugars and 8 g l^-1 ^protein were obtained in the low-cost media.

In all cases, the initial pH was adjusted to 6.7 and the media were sterilised at 121°C for 15 min. A glass 2 l-bioreactor with a working volume of 1.8 l was utilised for HA production under the following conditions: agitation at 500 rpm, no aeration, temperature maintained at 37°C and pH controlled with sterile NaOH (5 M).

### Sampling and analytical determinations

Samples from the bioreactor were initially blended with a 10% volume of 5% (w/v) SDS for 10 min. The biomass was separated by centrifugation at 5,000 g for 30 min and the sediment washed and resuspended in distilled water to the appropriate dilution for measuring the optical density (OD) at 700 nm. A calibration curve was used for determining the dry weight. The total sugars, reducing sugars, total amylolytic activity, lactic acid and proteins were measured in a first aliquot of supernatant. In a second aliquot of supernatant, HA was precipitated by mixing the supernatant with three volumes of ethanol and then centrifuging it at 5,000 g for 10 min. The sediment was redissolved with 1 volume of NaCl (1.5 M) and 3 volumes of ethanol and subsequently centrifuged at 5,000 g for 10 min. Finally, this last sediment was resuspended in distilled water for HA determination.

HA was analysed by the method developed by Blumenkrantz and Asboe-Hansen [45] following the proposal and mathematical corrections defined by Murado et al. [46]. Other analyses for media and samples (in duplicate) were: Total nitrogen: determined by the method of Havilah et al. [47]; Proteins: using the method of Lowry et al. [48]; Reducing sugars: the 3,5-dinitrosalicylic reaction [49]; Total sugars: the phenol-sulphuric reaction [50] according to the application developed by Strickland and Parsons [51] with glucose as a standard; Total amylolytic activity: the method described in Murado et al. [52]; LA: HPLC using an ION-300 column (Transgenomic, USA) with 6 mM sulphuric acid as the mobile phase (flow = 0.4 ml min^-1^) at 65°C and a refractive-index detector. The molecular weight (*M_w_*) of HA was determined by means of size-exclusion chromatography on HPLC equipped with an Ultrahydrogel linear column (Waters, USA) with 0.1 M NaNO_3 _as the mobile phase (flow = 0.6 ml min^-1^) and a refractive-index detector. Standards of polystyrene (Sigma) with different molecular weights (32, 77, 150, 330, 990 and 2600 kDa) were used for calibration.

### Mathematical models

In order to model the kinetic profiles of *S. zooepidemicus *(growth: *X*, HA: *H*, LA: *L *and carbohydrate-substrate consumption: *S*) and to obtain comparative production parameters, a set of reparameterised logistic equations and a Luedeking-Piret like equation were used [26,53,54]:(1)

where, *X *is the biomass production (g l^-1^), *X_m _*is the maximum biomass (g l^-1^), *v_x _*is the maximum growth rate (g l^-1 ^h^-1^) and *λ*_*x *_is the growth lag phase (h).(2)

where *H *is the HA production (g l^-1^), *H_m _*the HA production (g l^-1^), *v_h _*the maximum HA production rate (g l^-1 ^h^-1^) and *λ*_*h *_the HA lag phase (h).(3)

where, *L *is the LA production (g l^-1^), *L_m _*is the maximum LA production (g l^-1^), *v_l _*is the maximum LA production rate (g l^-1 ^h^-1^) and *λ*_*l *_is the LA lag phase (h).(4)

where *S *is the concentration of the substrate that is reducing sugars (RS) or total sugars (TS) (in g l^-1^), *S_0 _*the initial concentration of substrate (g l^-1^), *Y_x/s _*the biomass yield per substrate consumed (g of biomass g^-1 ^of substrate), *X_0 _*the initial biomass (g l^-1^) and *m_e _*the maintenance coefficient (g of substrate g^-1 ^of biomass h^-1^).

The production yields per biomass formed were obtained by means of the following equations [38]:(5)(6)

where *Y_h/x _*is the HA production yield per biomass formed (g of HA g^-1 ^of biomass) and *Y_l/x _*is the LA production yield per biomass formed (g of LA g^-1 ^of biomass).

### Numerical methods

Fitting procedures and parametric estimations calculated from the results were carried out by minimising the sum of quadratic differences between the observed and model-predicted values using the non-linear least-squares (Levenberg-Marquadt) method provided by DataFit 9.0.59 (Oakdale Engineering, USA). This software was also used to evaluate the significance of the parameters estimated by adjusting the experimental values to the proposed mathematical models (Student's t test with α = 0.05) and the consistency of these equations (Fisher's F test with α = 0.05).

## Competing interests

The authors declare that they have no competing interests.

## Authors' contributions

JAV performed the experiments, developed the mathematical models and wrote the manuscript. MIM and JF helped in the analytical determinations and helped in the design of the experiments. MAM has been involved in manuscript preparation and critical reading as well as in the design of the present study. All authors read and approved the manuscript.
